# Impact of F-18 Fluorodeoxyglucose PET/CT and PET/MRI on Initial Staging and Changes in Management of Pancreatic Ductal Adenocarcinoma: A Systemic Review and Meta-Analysis

**DOI:** 10.3390/diagnostics10110952

**Published:** 2020-11-14

**Authors:** Jeong Won Lee, Joo Hyun O, Miyoung Choi, Joon Young Choi

**Affiliations:** 1 Department of Nuclear Medicine, Catholic Kwandong University College of Medicine, International St. Mary’s Hospital, 25, Simgok-ro 100 Beon-gil, Seo-gu, Incheon 22711, Korea; sads00@naver.com; 2 Division of Nuclear Medicine, Department of Radiology, Seoul St. Mary’s Hospital, College of Medicine, The Catholic University of Korea, 222, Banpo-daero, Seocho-gu, Seoul 06591, Korea; ojoohyun@gmail.com; 3 National Evidence-Based Healthcare Collaborating Agency (NECA), 173, Toegyero, Jung-gu, Seoul 04554, Korea; myhams95@gmail.com; 4 Samsung Medical Center, Department of Nuclear Medicine, Sungkyunkwan University School of Medicine, 81, Irwon-ro, Gangnam-gu, Seoul 06351, Korea

**Keywords:** pancreatic carcinoma, fluorodeoxyglucose F-18, positron emission tomography, staging, meta-analysis

## Abstract

A systemic review and meta-analysis were conducted to investigate the diagnostic ability for staging and impact on management of F-18 fluorodeoxyglucose (FDG) positron emission tomography/computed tomography (PET/CT) and PET/magnetic resonance imaging (MRI) in patients with pancreatic ductal adenocarcinoma. A comprehensive search was performed in four databases to retrieve studies of pancreatic ductal adenocarcinoma patients that have reported the diagnostic ability of FDG PET/CT and PET/MRI for detecting metastasis and the proportion of patients whose management was changed by its results. The sensitivity and specificity for detecting metastasis and the proportion of patients with management changes were pooled using a random-effects model. A total of 10 studies were included. The pooled sensitivity and specificity for detecting lymph node metastasis were 0.55 and 0.94, respectively, while the pooled sensitivity and specificity for detecting distant metastasis were 0.80 and 1.00, respectively. The areas under the summarized receiver operating characteristic curves for detecting lymph node and distant metastasis were 0.88 and 0.92, respectively. The pooled proportion of patients with management changes was 19%. FDG PET/CT and PET/MRI showed high diagnostic accuracy for detecting lymph node and distant metastasis in pancreatic ductal adenocarcinoma patients, and the use of these imaging tools led to management changes in a significant portion of these patients.

## 1. Introduction

Pancreatic cancer is notorious for its extremely poor prognosis [1]. A previous study reported the 5-year survival rate of pancreatic cancer to be only 9%, and it is the seventh leading cause of cancer death in both men and women worldwide [1,2]. The only potential curative treatment for pancreatic cancer is radical surgical resection [3]. However, according to several recent reviews, at the time of initial staging work-up, distant metastatic lesions are found in over 50% of pancreatic cancer patients and only 20% of patients have resectable disease [1,3]. In order to choose the most suitable treatment and to prevent unnecessary surgery, accurate tumor staging is essential for patients with pancreatic cancer [3,4]. Therefore, various anatomical imaging modalities including contrast-enhanced computed tomography (CT), magnetic resonance imaging (MRI), and endoscopic ultrasonography are used in the initial staging work-up of pancreatic cancer [5].

In addition to anatomical imaging examinations, positron emission tomography (PET) imaging using F-18 fluorodeoxyglucose (FDG), a radiotracer preferentially taken up by malignant cells, is currently used for the evaluation of pancreatic cancer [5]. In previous studies of pancreatic cancer, FDG PET showed significant clinical value for differentiating malignant pancreatic lesions from benign lesions, detecting metastatic lesions, predicting prognosis after treatment, and assessing cancer recurrence [6,7,8,9,10]. Considering the high prevalence of distant metastasis in patients with pancreatic cancer, it is reasonable to assume that FDG PET could play a significant role in the staging work-up of pancreatic cancer; however, there remain controversies in defining its roll [11,12,13,14,15]. Furthermore, previous meta-analyses of the role of FDG PET in staging pancreatic cancer consisted mainly of studies that used a PET scanner [4,16]. With the advancement of medical technology, a dedicated PET/CT or PET/MRI scanner is more commonly used in clinical practice currently, rather than a PET scanner [5,17]. In FDG PET images, several intra-abdominal organs, such as the bowel and liver, can exhibit physiological FDG uptake, which can lead to uncertain diagnoses and high false-positive rates in cases using interpretation of stand-alone PET images for staging [5]. On the other hand, PET/CT and PET/MRI are known to improve lesion identification and localization with the aid of anatomical information provided by CT and MRI images, thereby increasing diagnostic accuracy [5,18]. Therefore, a comprehensive review of studies using FDG PET/CT and PET/MRI, rather than PET alone, is required to establish their role for staging pancreatic cancer in current clinical practice.

In the present study, we systematically reviewed the available literature and performed a meta-analysis to investigate the diagnostic ability of staging FDG PET/CT and PET/MRI for detecting lymph node and distant metastasis in patients with pancreatic ductal adenocarcinoma. We also conducted a meta-analysis of the impact of staging FDG PET/CT and PET/MRI on management in those patients.

## 2. Materials and Methods

The methodological approach of this meta-analysis complied with the Preferred Reporting Items for Systematic Reviews and Meta-Analyses (PRISMA) of Diagnostic Test Accuracy Studies [19]. All processes regarding study eligibility assessment, data extraction, and methodological quality assessment were independently and repeatedly performed by two independent reviewers (J.W.L., J.Y.C.). Disagreement was resolved via discussion and consensus among the reviewers.

### 2.1. Literature Search

A systematic literature search was performed in four different bibliographic databases (PubMed, Embase, Cochrane Library, and KoreaMed) for all studies published from 1 January 2000 to 6 November 2019 using combinations of the following keywords: “pancreatic neoplasm” or “pancreas tumor” or “pancreas cancer” or “pancreas carcinoma” or “pancreas adenocarcinoma” or “pancreas malignancy” AND “positron emission tomography” or “PET” or “PET/CT” or “PET-CT” or “PET/MRI” or “PET-MRI” AND “staging” or “stage” or “diagnosis” or “detect” or “metastasis” AND “diagnostic accuracy” or “sensitivity” or “specificity” or “receiver operating characteristic” or “ROC curve” or “area under the curve” or “AUC” or “added value” or “diagnostic yield” or “predict” or “accuracy”. No language restriction was applied for the literature search. In addition, reference lists of retrieved studies were also screened to avoid missing eligible studies.

### 2.2. Inclusion and Exclusion Criteria

All relevant literature underwent evaluation for eligibility according to inclusion criteria based on the patient/intervention/comparator/outcome/study (PICOS) approach [20], as follows: (1) individuals with newly diagnosed pancreatic ductal adenocarcinoma as the patients; (2) FDG PET/CT or PET/MRI as the intervention; (3) no comparator; (4) the diagnostic performance for staging pancreatic cancer, confirmed by histopathological evaluation or imaging studies, and the proportion of patients with management changes as the outcomes; and (5) original articles as the study type. The exclusion criteria were as follows: studies that (1) enrolled any patients with malignant pancreatic diseases other than pancreatic ductal adenocarcinoma, such as neuroendocrine tumors, metastasis, or lymphoma; (2) used PET, not PET/CT, or PET/MRI as the intervention; (3) provided insufficient information for calculating true-positive, false-positive, true-negative, and false-negative rates for estimating diagnostic performance during per-patient analysis or for calculating the proportion of patients with management changes; (4) were publication types other than original articles, including case reports, reviews, letters, editorials, and conference abstracts; (5) were in vitro or animal studies; (6) had study populations that overlapped with other literature; (7) were published in languages other than English or Korean; or (8) were not available in full text.

### 2.3. Data Extraction and Quality Assessment

The following information was extracted from each of the included studies: the first author, publication year, country, study design, number and characteristics (age, sex, and enrollment criteria) of patients, details of the intervention techniques (imaging modality and imaging analytical method), reference standard, and outcomes. The methodological quality of the included studies was assessed using the Quality Assessment of Diagnostic Accuracy Studies (QUADAS)-2 tool [21]. The QUADAS-2 tool consists of 14 questions with 4 key domains—patient selection, index test, reference standard, and flow and timing. Each question was answered with “yes” for a low risk of bias, “no” for a high risk of bias, and “unclear” if insufficient data were reported [21].

### 2.4. Statistical Analysis

The primary outcome of this meta-analysis was the diagnostic accuracies of FDG PET/CT and PET/MRI for detecting lymph node metastasis (N staging) and distant metastasis (M staging) in patients with pancreatic cancer. The secondary outcome was the impact of FDG PET/CT and PET/MRI on patient management, as assessed by calculating the proportion of patients whose therapeutic plan was changed due to the PET/CT or PET/MRI findings.

With the data extracted from the included studies, the pooled sensitivity, specificity, positive likelihood ratio (PLR), negative likelihood ratio (NLR), and diagnostic odds ratio (DOR) in detecting lymph node metastasis and distant metastasis were calculated by using a bivariate meta-analysis method with a random-effects model. Furthermore, summarized receiver operating characteristic (SROC) curves were generated for evaluating the diagnostic abilities of PET/CT and PET/MRI using the calculated area under the curves (AUC). The proportion of patients with management changes was calculated for each study, and the results were meta-analytically pooled using a random-effects model. The I^2^ statistic was used to assess the heterogeneity among the included studies. An I^2^ value lies from 0% to 100%, with a value of >50% suggesting substantial heterogeneity [22]. In meta-analyses with an I^2^ > 50%, subgroup analyses were further performed to investigate the sources of heterogeneity; investigated subgroups were country (Asia vs. non-Asia), study design (prospective vs. retrospective), and analytical method of PET images (qualitative analysis vs. quantitative analysis). Publication bias was assessed using Deek’s funnel plot and Egger’s test. All statistical analyses were performed using Stata software version 15.0 (StataCorp, College Station, TX, USA).

## 3. Results

### 3.1. Study Selection and Characteristics

**Figure 1:**
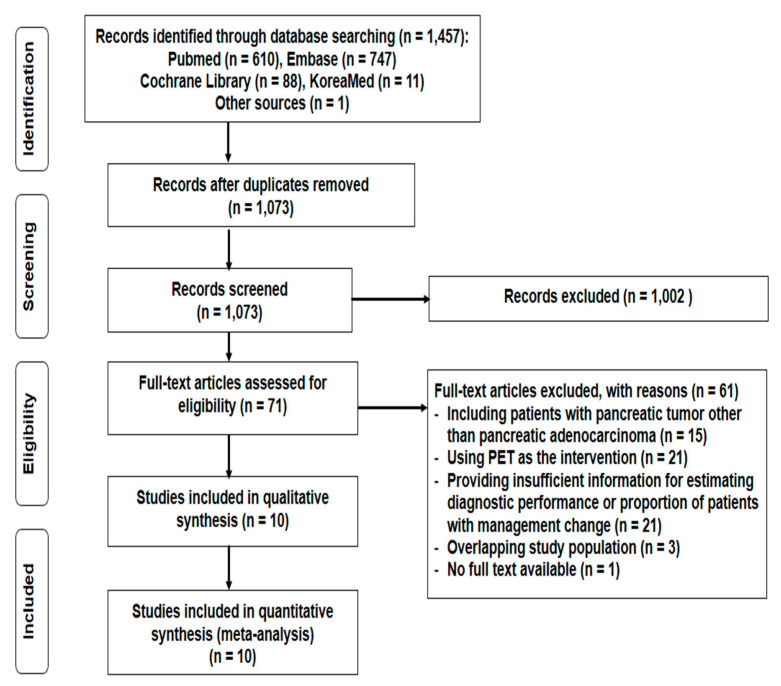
PRISMA flow diagram showing the process of study selection.

The process of study selection is depicted in Figure 1. In the systematic literature search, a total of 1457 articles were initially retrieved (Supplementary Table S1). After excluding 384 duplicated articles and 1002 articles based on title and abstract screening, 71 articles were potentially eligible. On full-text assessment for eligibility, 61 studies were excluded; hence, 10 studies comprising 852 patients were finally included in our meta-analysis.

The characteristics of the 10 enrolled studies are shown in Table 1. All included studies were published in English; three studies were prospective [14,15,23], while the remaining seven were retrospective [6,11,12,13,24,25,26]. The number of enrolled patients in each study ranged from 37 to 261, with two studies showing enrollment of >100 patients [11,15]. Of the 10 included studies, only one was a multi-center study [15], while the remaining were single-center studies. As for imaging methods, one study evaluated the diagnostic performance of PET/MRI [23], while in all other included studies the diagnostic ability of PET/CT was assessed. For the imaging analytical method, six studies used visual assessment for determining lesions with positive FDG uptake [6,14,23,24,25,26], whereas four studies performed quantitative analysis using the cut-off standardized uptake value (SUV) of 2.5 [11,13], 3.0 [12], or 3.5 [15] for determining malignant involvement.

### 3.2. Quality Assessment

**Figure 2:**
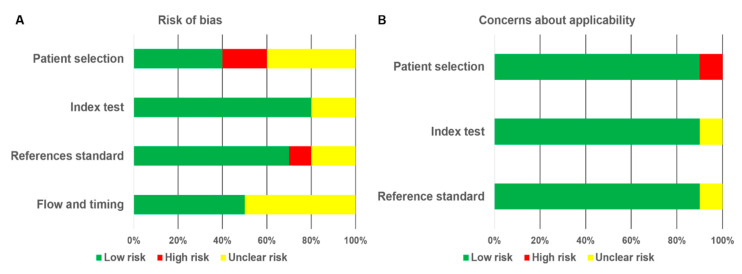
Quality assessment of 10 included studies using the QUADAS-2 tool to evaluate the risk of bias (**A**) and concerns about applicability (**B**). Each diagram shows the percentage of studies with low (green), high (red), and unclear (yellow) risk of bias.

The methodological quality assessment of the included studies is shown in Figure 2 (Supplementary Table S2). In the domain of patient selection, six studies were judged to have high or unclear risk of bias, which was mainly due to providing insufficient information regarding consecutive enrollment of patients [6,11,13,24,25,26]. For the reference standard, three studies were judged to have high or unclear risk of bias because the blinding method was not used in interpreting the references standard results [11,23,26]. For the flow and timing, the major weaknesses were the lack of information regarding the interval between the index tests and reference standard and the lack of a uniform reference test for enrolled patients. Meanwhile, only a single study was judged to be high risk with regard to the concerns about applicability in the domain of patient selection [6]. Furthermore, a single study was considered to have unclear risk with regard to applicability concerns in both the index test [11] and reference standard [23] domains.

### 3.3. Diagnostic Performance of PET/CT and PET/MRI for Detection of Lymph Node and Distant Metastases

**Figure 3:**
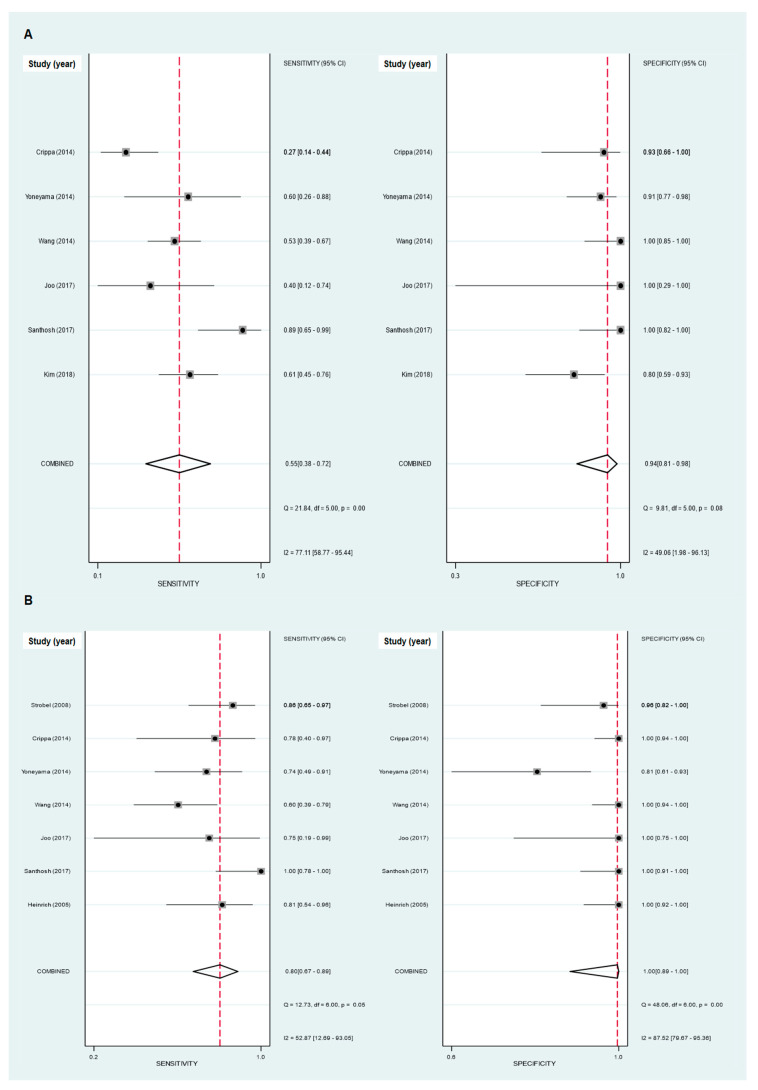
Forest plots of the pooled sensitivity and specificity for detecting lymph node metastasis (**A**) and for detecting distant metastasis (**B**), with 95% confidence intervals. Each solid square represents the sensitivity and specificity of each of the included studies, and the diamond indicates the pooled sensitivity and specificity.

**Figure 4:**
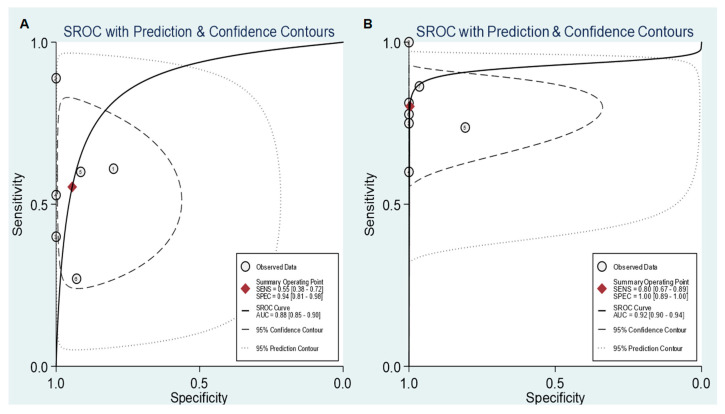
Summarized receiver operating characteristic (SROC) curves of FDG PET/CT and PET/MRI for detecting lymph node metastasis (**A**) and distant metastasis (**B**). Each circle represents the sensitivity and specificity of each of the included studies. The diamond is the summary point for the average sensitivity and specificity estimates. The ellipse with the dashed line is the 95% confidence region, and the ellipse with the dotted line is the 95% prediction region.

Six studies with a total of 357 patients with pancreatic ductal adenocarcinoma were included in the meta-analysis of using FDG PET/CT and PET/MRI for diagnosing lymph node metastasis [6,12,13,23,24,26]. The pooled results from these studies demonstrated that the sensitivity and specificity of FDG PET/CT and PET/MRI for detecting lymph node metastasis were 0.55 (95% confidence interval [CI]): 0.38–0.72) and 0.94 (95% CI: 0.81–0.98), respectively (Figure 3A). Furthermore, the pooled PLR, NLR, and DOR were 9.87 (95% CI: 2.55–38.25), 0.47 (95% CI: 0.31–0.72), and 2.11 (95% CI: 1.40–3.20), respectively, with an AUC of SROC of 0.88 (95% CI: 0.85–0.90; Figure 4A). Of the six included studies, one study evaluated the diagnostic ability of PET/MRI [23], which showed a similar sensitivity (0.40; 95% CI: 0.12–0.74) and specificity (1.00; 95% CI: 0.29–1.00) to those obtained from the studies evaluating PET/CT.

Seven studies with a total of 296 patients provided data regarding the diagnostic ability of FDG PET/CT and PET/MRI for detecting distant metastasis [6,12,13,14,23,25,26]. The pooled sensitivity and specificity of FDG PET/CT and PET/MRI for diagnosing distant metastasis were 0.80 (95% CI: 0.67–0.89) and 1.00 (95% CI: 0.89–1.00), respectively (Figure 3B). The pooled PLR, NLR, and DOR were 215.30 (95% CI: 7.39–6273.09), 0.20 (95% CI: 0.12–0.34), and 1084.20 (95% CI: 33.92–34,658.01), respectively, with an AUC of SROC of 0.92 (95% CI: 0.90–0.94; Figure 4B). The study which evaluated PET/MRI [23] found a sensitivity and specificity of 0.75 (95% CI: 0.19–0.99) and 1.00 (95% CI: 0.75–1.00), respectively, for detecting distant metastasis, which were similar to the results of other studies evaluating PET/CT.

### 3.4. Management Changes Following PET/CT

**Figure 5:**
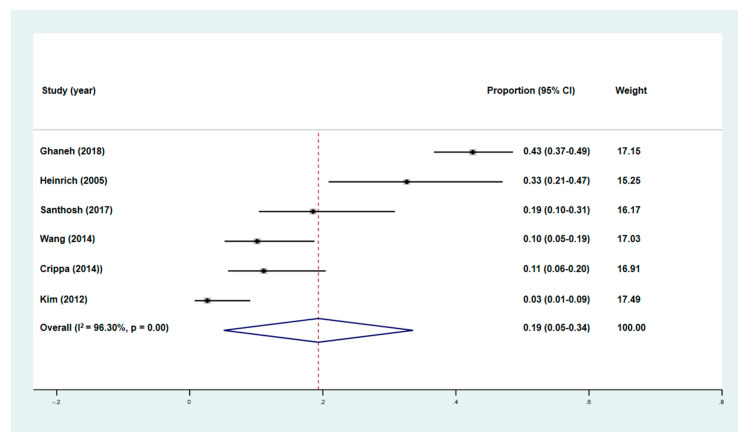
Forest plot of the pooled proportion of patients who underwent management changes after FDG PET/CR and PET/MRI, with 95% confidence intervals. Each solid square represents the proportion of patients who underwent management changes in each individual study, while the diamond represents the pooled proportion.

Six studies with a total of 650 patients assessed the proportions of patients who underwent management changes following FDG PET/CT staging and compared it to that of conventional staging [6,11,12,13,14,15]. All six studies performed PET/CT for initial staging of pancreatic cancer. The pooled percentage of patients who underwent management changes following FDG PET/CT was 19% (95% CI: 5–34%, Figure 5). In all six studies, the most common reason of management change was identifying unknown metastatic lesions by PET/CT. Additionally, PET/CT also changed the management plan by detecting secondary primary malignancies and ruling out metastasis of lesions found on other imaging modalities.

Among the six studies, two prospective studies have evaluated the cost-effectiveness of PET/CT in staging of pancreatic cancer [14,15]. In a study by Heinrich et al. [14], PET/CT was cost saving by avoiding patients from unnecessary surgery, showing cost-savings of USD 1066 per patient. In another study by Ghaneh et al. [15], PET/CT was both less costly and more effective when compared to contrast-enhanced CT. A subgroup of pancreatic cancer patients with resection showed the most cost-effective results, showing cost-savings of GBP 1542 per patient.

### 3.5. Heterogeneity Analysis

**Figure 6:**
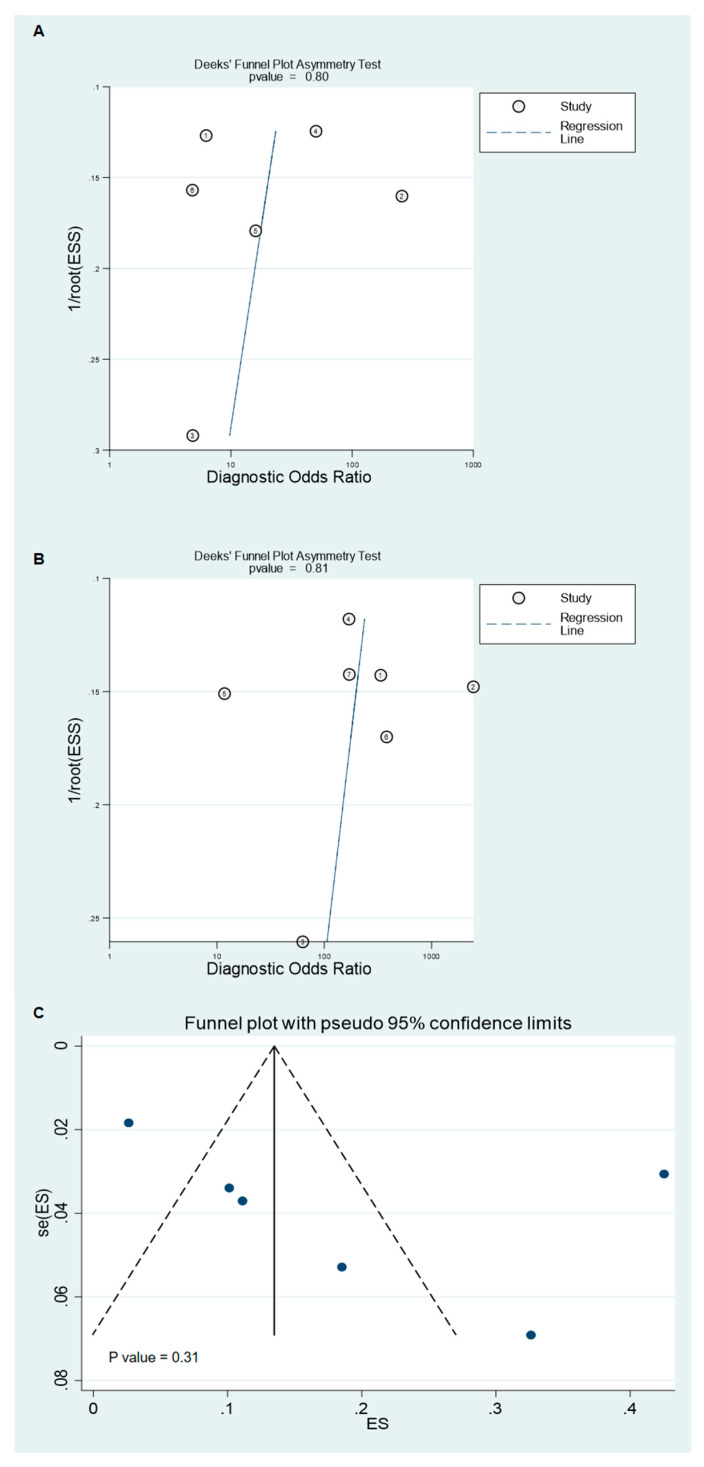
Funnel plots of studies evaluating the diagnostic ability of FDG PET/CT and PET/MRI for detecting lymph node metastasis (**A**), studies evaluating the diagnostic ability of FDG PET/CT and PET/MRI for detecting distant metastasis (**B**), and studies evaluating the proportion of patients who underwent management changes following imaging (**C**).

There was substantial heterogeneity in the sensitivity for lymph node metastasis (*p* < 0.001; I^2^ = 77.1; 95% CI: 58.8–95.4%; Figure 3A), the specificity for distant metastasis (*p* < 0.001; I^2^ = 87.5; 95% CI: 79.7–95.4%; Figure 3B), and the proportion of patients who underwent management changes following FDG PET/CT staging ((*p* < 0.001; I^2^ = 96.3; 95% CI: 91.1–98.7%; Figure 5) among the included studies. The specificity for lymph node metastasis (*p* = 0.08; I^2^ = 49.1%; 95% CI: 2.0–96.1%; Figure 3A) and the sensitivity for distant metastasis (*p* = 0.05; I^2^ = 52.9%; 95% CI: 12.7–93.1%; Figure 3B) showed borderline significance with regard to the I^2^ statistic. No significant publication bias was shown for all meta-analyses in the current study (*p* = 0.80 for diagnostic ability in detecting lymph node metastasis; *p* = 0.81 for diagnostic ability in detecting distant metastasis; *p* = 0.31 for proportion of patients who underwent management changes; Figure 6).

### 3.6. Subgroup Analysis

Subgroup analyses was performed based on the country, study design, and the analytical method of each study (Table 2). For the diagnostic ability of FDG PET/CT and PET/MRI for lymph node metastasis, because there was only one study performed in a non-Asian country and only one with a prospective study design, we performed a subgroup analysis only based on the analytical method. On this subgroup analysis, studies that used qualitative analytical methods showed a higher sensitivity for detecting both lymph node metastasis (0.64 vs. 0.40, respectively) and distant metastasis (0.84 vs. 0.64, respectively) than those that used quantitative analytical methods (*p* < 0.05 for both). Meanwhile, all analyzed factors failed to explain the heterogeneity in the specificity for detecting lymph node and distant metastasis. In subgroup analysis of the proportion of subjects who underwent management changes, studies in non-Asian countries, with a prospective study design, and with qualitative analytical methods revealed higher proportions of patients with management changes following PET/CT (28.2 vs. 9.8%, respectively, for country; 39.9 vs. 10.2%, respectively, for study design; 25.5 vs. 14.8%, respectively, for analytical method) than those in Asian countries, with a retrospective design, and with quantitative analytical methods (*p* < 0.05 for all).

## 4. Discussion

In patients with pancreatic cancer, contrast-enhanced CT is the preferred primary imaging modality for the initial evaluation [27]. Additionally, MRI and endoscopic ultrasound have been commonly used to delineate primary tumors, to evaluate blood vessel involvement, and to detect metastatic lesions [27,28]. Although most pancreatic cancer lesions showed increased FDG uptake, the potential benefits of FDG PET/CT in staging pancreatic cancer remains contentious [9,27,29]. In previous studies, staging FDG PET/CT showed a high diagnostic accuracy for detecting metastatic lesions of pancreatic cancer [13,14,25,26], and it has been recommended in patients with localized disease for detecting metastatic lesions in guidelines from the Japan Pancreas Society, the United Kingdom National Institute for Health and Care Excellence, and the National Comprehensive Cancer Network [30,31,32]. By contrast, because of the relatively small proportion of patients in whom additional metastatic lesions were found only by FDG PET/CT, other studies have suggested that PET/CT has a limited role in the staging work-up of pancreatic cancer [11,12]. In guidelines from the American Society of Clinical Oncology and the European Society for Medical Oncology, the use of FDG PET/CT is not routinely recommended for the management of pancreatic cancer patients [28,33]. In the present meta-analysis, FDG PET/CT and PET/MRI showed only moderate sensitivity for detecting lymph node metastasis. Meanwhile, PET/CT and PET/MRI demonstrated high specificity for detecting lymph node metastasis and high sensitivity and specificity for detecting distant metastasis, showing high AUC of the SROC values for both lymph node and distant metastasis. Furthermore, the findings from FDG PET/CT and PET/MRI led to management changes in 19% of pancreatic cancer patients mainly by identifying unknown metastatic lesions, and PET/CT was cost-effective for pancreatic cancer staging with cost-savings of more than USD 1000 per patient. The results of our study suggest that FDG PET/CT and PET/MRI could be a valuable diagnostic imaging modality for initial staging in patients with pancreatic cancer and could have a significant impact on determining therapeutic plans for these patients. Considering that, in the included studies, FDG PET/CT and PET/MRI both had a high diagnostic accuracy for distant metastasis, and most patients who underwent management changes following FDG PET/CT or PET/MRI were upstaged to stage IV [6,12,13,14,15,26], FDG PET/CT and PET/MRI should be routinely recommended in patients with localized pancreatic cancer who plan to receive curative treatment.

In previous meta-analyses of studies of pancreatic cancer patients, the sensitivity and specificity of FDG PET and PET/CT were 32–67% and 75–81%, respectively, for identifying lymph node metastasis and 57–67% and 96–100%, respectively, for distant metastasis [4,34]. Since those meta-analyses mainly included studies that utilized a PET scanner or studies that enrolled patients with malignant pancreatic diseases other than pancreatic ductal adenocarcinoma [4,16,34], it is difficult to make a direct comparison of our results with the results of these previous meta-analyses. However, our results demonstrated higher specificity for detecting lymph node metastasis and higher sensitivity for detecting distant metastasis with FDG PET/CT or PET/MRI than the results of those previous meta-analyses [4,34]. Given that anatomical information gleaned from CT images from PET/CT scans can increase the specificity for lymph node staging and the sensitivity for detecting metastatic lesions [5,18,25,35], these results may suggest an incremental increase in the diagnostic value of PET/CT and PET/MRI compared with PET alone.

Our meta-analyses found substantial heterogeneity across the included studies. Notably, in subgroup analyses performed to investigate the source of this heterogeneity, the method of analyzing PET images was found to be a significant factor for heterogeneity in all meta-analyses. Qualitative analysis is subjective and known to be dependent on the clinical experience of the reader, which might lead to significant inter-reader discrepancies [36,37,38]. Hence, quantitative analysis using SUV has been widely used for objective assessment of malignant diseases and has shown comparable results to those of qualitative analysis [36,37,38]. However, the results of our subgroup analysis revealed that studies that used qualitative analytical methods showed significantly higher sensitivity for lymph node and distant metastasis and a higher proportion of patients with management changes than studies using quantitative methods. This might be due to the different cut-off values used in the individual studies that utilized quantitative analysis, as there is no determined cut-off SUV for defining metastatic lesions [36]. Further studies would be needed to clarify the effects of analytical methods on PET/CT and PET/MRI results. Another potential source of the heterogeneity between studies for the proportion of subjects who underwent management changes was study design. In the subgroup analysis, the prospective studies had significantly higher proportions of patients with management changes than the retrospective ones. Given that retrospective studies use the data that might be collected for another objective and are often assumed to have more bias than prospective ones [39], this result might imply the underestimation of the effect of FDG PET/CT on therapeutic planning in the retrospective studies. Additionally, variations in the country where the studies were performed was another potential cause of heterogeneity seen between studies for the proportion of patients who underwent management changes.

The present analysis had several limitations that need to be addressed. First, the number of studies included in this meta-analysis was relatively small, and most of the included studies were retrospectively performed (7 out of 10 studies) or single-center studies (9 out of 10 studies). Further studies are necessary which include a large number of prospective and multi-center studies. Second, the definition of a positive lesion on PET/CT, as well as reference standards, varied among the included studies which could affect the accuracy of the results. Third, due to insufficient information regarding the stages of those patients who underwent management changes following PET/CT, the pooled proportion of patients with management changes could not be stratified by initial stage. However, given this limitation, our results could support the clinical use of PET/CT in pancreatic cancer irrespective of the initial stage. Fourth, the definition of changes in patient management following PET/CT staging might differ among the enrolled studies. Furthermore, although the diagnostic performance of PET/MRI was similar to that of PET/CT, because only a single study evaluated the diagnostic performance of PET/MRI [23], we could not perform subgroup analysis to compare diagnostic ability between PET/MRI and PET/CT. Lastly, there was substantial heterogeneity across the studies, and we were unable to find the potential source for the heterogeneity in specificity. Hence, the general application of our pooled results might be limited to specific clinical conditions.

## 5. Conclusions

In the present meta-analysis, FDG PET/CT and PET/MRI showed high specificity for detecting lymph node metastasis and high sensitivity and specificity for identifying distant metastasis in patients with pancreatic ductal adenocarcinoma. Furthermore, FDG PET/CT and PET/MRI had a significant impact on the management of pancreatic ductal adenocarcinoma, with our results showing a pooled proportion of 19% of patients who underwent management changes following imaging. Based on our results, FDG PET/CT and PET/MRI would be considered to be one of the routine imaging examinations used in the staging work-up of pancreatic cancer. However, because of the relatively small number of included studies with substantial heterogeneity among them, further prospective studies with larger populations are needed to further elucidate our results.

## Figures and Tables

**Table 1:** The characteristics of included studies.

First Author/Year	Country	Study Design	No. of Patients	Median Age (Years)	Inclusion Criteria	Imaging Method	FDG Uptake Time (min)	Analytical Method	Reference Standard
Crippa/2014	Italy	Retrospective	72	65	Potentially resectable pancreatic cancer	PET/CT	60	QN	Histopathological confirmation
Ghaneh/2018	UK	Prospective	261	66	Staging work-up for suspected pancreatic cancer	PET/CT	90	QN	Histopathological confirmation and follow-up imaging studies
Heinrich/2005	Switzerland	Prospective	59	61	Staging work-up for suspected pancreatic cancer	PET/CT	60	QL	Histopathological confirmation and imaging-based decisions
Joo/2017	Korea	Prospective	37	63 *	Potentially resectable pancreatic cancer	PET/MRI	50–90	QL	Histopathological confirmation and imaging-based decisions
Kim/2012	Korea	Retrospective	125	62	Staging work-up for histopathologically proven pancreatic cancer	PET/CT	45	QN	Histopathological confirmation and follow-up imaging studies
Kim/2018	Korea	Retrospective	70	69	Pancreatic cancer patients with radical surgery	PET/CT	60–70	QL	Histopathological confirmation
Santhosh/2017	India	Retrospective	54	58 *	Staging work-up for histopathologically proven pancreatic cancer	PET/CT	60 ± 10	QL	Histopathological confirmation and imaging-based decisions
Strobel/2008	Switzerland	Retrospective	50	64 *	Staging work-up for histopathologically proven pancreatic cancer	PET/CT	60	QL	Histopathological confirmation and follow-up imaging studies
Wang/2014	China	Retrospective	79	63 *	Staging work-up for histopathologically proven pancreatic cancer	PET/CT	NS	QN	Histopathological confirmation
Yoneyama/2014	Japan	Retrospective	45	67 *	Staging work-up for suspected pancreatic cancer	PET/CT	63 ± 5	QL	Histopathological confirmation and follow-up imaging studies

FDG, F-18 fluorodeoxyglucose; NS, not specified; PET/CT, positron emission tomography/computed tomography; PET/MRI, positron emission tomography/magnetic resonance imaging; QL, qualitative analysis; QN, quantitative analysis; UK, the United Kingdom. * mean age.

**Table 2:** The results of subgroup analysis.

Factors	Lymph Node Metastasis			Distant Metastasis			Management Change	
	No. of Studies	Sensitivity (95% CI)	Specificity (95% CI)	No. of Studies	Sensitivity (95% CI)	Specificity (95% CI)	No. of Studies	Proportion (95% CI)
Country								
Asia				4	0.78 (0.54–0.95)	0.97 (0.87–1.00)	3	9.8 * (2.8–20.4)
Non-Asia				3	0.81 (0.69–0.91)	0.99 (0.96–1.00)	3	28.2 * (10.4–50.5)
Study design								
Prospective				2	0.78 (0.58–0.92)	0.99 (0.95–1.00)	2	39.9 * (31.5–48.5)
Retrospective				5	0.80 (0.63–0.93)	0.97 (0.91–1.00)	4	10.2 * (4.7–17.5)
Analytical method								
Qualitative	4	0.64 * (0.45–0.81)	0.91 (0.80–0.98)	5	0.84 * (0.71–0.94)	0.96 (0.89–1.00)	2	25.5 * (13.3–40.1)
Quantitative	2	0.40 * (0.18–0.65)	0.96 (0.85–1.00)	2	0.64 * (0.48–0.79)	1.00 (0.98–1.00)	4	14.8 * (1.6–37.8)

CI, confidence interval. * Significant difference between these subgroups (*p* < 0.05).

## Supplementary Materials

The following are available online at https://www.mdpi.com/2075-4418/10/11/952/s1, Table S1: The queries and results of electronic searches in the PubMed, Embase, Cochrane Library, and KoreaMed databases; Table S2: Quality assessment of included studies using the QUADAS-2 tool.
