# Supplementary material for: Impact of F-18 Fluorodeoxyglucose PET/CT and PET/MRI on Initial Staging and Changes in Management of Pancreatic Ductal Adenocarcinoma: A Systemic Review and Meta-Analysis
Source: Diagnostics (Basel). 2020 Nov 14;10(11):952. doi: 10.3390/diagnostics10110952 (PMC7696716; doi:10.3390/diagnostics10110952)
Supplement: Supplementary file 1 [file diagnostics-10-00952-s001.pdf]

Supplementary Table S1. The queries and results of electronic searches in the PubMed, Embase, Cochrane Library, and KoreaMed databases

PubMed

Date: 2019.11.06			
PICOS	#	Search queries	Articles #
P	1	exp Pancreatic Neoplasms/ OR (pancrea$ AND (tumo*r OR cancer OR carcinoma OR adenocarcinoma OR malignan$ OR neoplasm$ OR cyst)).tw.kw	115646
P	2	(staging OR stage OR diagnosis OR detect$ OR metastas$).tw,kw	4266478
P	3	1 AND 2	46238
I	4	exp Positron-Emission Tomography/ OR (Positron-Emission Tomography OR Positron Emission Tomography OR PET OR PET?CT OR PET-CT OR PET?MRI OR PET-MRI).tw,kw	120459
P&I	5	3 AND 4	1689
O	6	exp "Sensitivity and Specificity"/ OR (diagnos$ accuracy OR sensitivity OR specificity OR Receiver operating characteristic OR ROC curve OR Area under the curve OR AUC OR added value OR diagnostic yield OR prognosis OR predict$ OR accuracy).tw,kw	2937041
Limit	7	5 AND 6	703
Case report	8	case report$.pt	2052847
Final	9	7 not 8	649
Time	10	limit 9 to yr="2000 -Current"	610

Embase

Date: 2019.11.06			
PICOS	#	Search queries	Articles #
P	1	'pancreas tumor'/mj OR (pancrea* AND (tumo*r OR cancer OR carcinoma OR adenocarcinoma OR malignan* OR neoplasm* OR cyst)):ab,ti,kw	157859
P	2	(staging OR stage OR diagnosis OR detect* OR metastas*):ab,ti,kw	3854486
P	3	#1 AND #2	73184
I	4	'positron emission tomography'/exp OR (‘Positron-Emission Tomography’ OR ‘Positron Emission Tomography’ OR PET OR ‘PET CT’ OR ‘PET-CT’ OR ‘PET MRI’ OR ‘PET-MRI’):ab,ti,kw	233809
P&I	5	#3 AND #4	3719
O	6	'sensitivity and specificity'/exp OR (‘diagnos* accuracy’ OR sensitivity	2937041

		OR specificity OR 'Receiver operating characteristic' OR 'ROC curve' OR 'Area under the curve' OR AUC OR 'added value' OR 'diagnostic yield' OR prognosis OR predict* OR accuracy):ab,ti,kw	
Limit	7	5 AND 6	1455
Final	8	#7 AND ('article'/it OR 'article in press'/it OR 'review'/it)	778
Time	9	#8 AND [2000-2019]/py	747

Cochrane Library

Date: 2019.11.06			
PICOS	#	Search queries	Articles #
P	1	(pancrea* AND (tumo*r OR cancer OR carcinoma OR adenocarcinoma OR malignan* OR neoplasm* OR cyst))	7792
P	2	MeSH descriptor: [Pancreatic Neoplasms] explode all trees	1546
P	3	#1 OR #2	7801
P	4	(staging OR stage OR diagnosis OR detect* OR metastas*)	292139
P	5	#3 AND #4	3568
I	6	('Positron-Emission Tomography' OR 'Positron Emission Tomography' OR PET OR 'PET CT' OR 'PET-CT' OR 'PET MRI' OR 'PET-MRI')	8206
I	7	MeSH descriptor: [Positron-Emission Tomography] explode all trees	1018
I	8	#6 OR #7	8206
P&I	9	#5 AND #8	155
O	10	('diagnos* accuracy' OR sensitivity OR specificity OR 'Receiver operating characteristic' OR 'ROC curve' OR 'Area under the curve' OR AUC OR 'added value' OR 'diagnostic yield' OR prognosis OR predict* OR accuracy)	259885
O	11	MESH sensitivity and specificity	16229
O	12	#10 OR #11	259955
Limit	13	#9 AND 12	90
Final	14	#13 AND Cochrane Review, Trial<br>with Cochrane Library publication date from Jan 2000 to Nov 2019	88

KoreaMed

Date: 2019.11.06			
PICOS	#	Search queries	Articles #
	2	((staging OR stage OR (MeSH_Terms:"Diagnosis" OR All_Fields:"Diagnosis") OR detect* OR metastas*) :) AND ((pancrea* AND	11

		(tumo*r OR (MeSH_Terms:"Neoplasms" OR All_Fields:"Neoplasms" OR All_Fields:"cancer") OR (MeSH_Terms:"Carcinoma" OR All_Fields:"Carcinoma") OR (MeSH_Terms:"Adenocarcinoma" OR All_Fields:"Adenocarcinoma") OR malignan* OR neoplasm* OR (MeSH_Terms:"Cysts" OR All_Fields:"Cysts" OR All_Fields:"cyst"))) :) AND ('Positron-Emission Tomography' OR 'Positron Emission Tomography' OR PET OR 'PET CT' OR 'PET-CT' OR 'PET MRI' OR 'PET-MRI') :	

Supplementary Table S2. Quality assessment of included studies using the QUADAS-2 tool.

First author (year)	Risk of bias				Applicability concerns		
	Patient selection	Index test	Reference standard	Flow and timing	Patient selection	Index test	Reference standard
Crippa (2014)	Low	Unclear	Low	Low	Low	Low	Low
Ghaneh (2018)	Low	Low	Low	Low	Low	Low	Low
Heinrich (2005)	Low	Low	Low	Unclear	Low	Low	Low
Joo (2017)	Low	Low	Unclear	Low	Low	Low	Unclear
Kim (2012)	Unclear	Unclear	High	Unclear	Low	Unclear	Low
Kim (2018)	Unclear	Low	Low	Low	Low	Low	Low
Santhosh (2017)	High	Low	Low	Unclear	High	Low	Low
Strobel (2008)	High	Low	Low	Unclear	Low	Low	Low
Wang (2014)	Unclear	Low	Low	Low	Low	Low	Low
Yoneyama (2014)	Unclear	Low	Unclear	Unclear	Low	Low	Low

High, high risk; Low, low risk; QUADAS-2, the Quality Assessment of Diagnostic Accuracy Studies-2; Unclear, unclear risk
